# How can big data shape the field of non-religion studies? And why does it matter?

**DOI:** 10.1016/j.patter.2021.100263

**Published:** 2021-06-11

**Authors:** Dominik Balazka, Dick Houtman, Bruno Lepri

**Affiliations:** 1 University of Milan, Department of Social and Political Sciences, Milan, Lombardy 20122, Italy; 2 KU Leuven, Center for Sociological Research, Faculty of Social Sciences, Leuven, Flemish Brabant 3000, Belgium; 3 Bruno Kessler Foundation, Mobile and Social Computing Lab, Trento, Trentino-Alto Adige 38100, Italy; 4 University of Turin, Department. of Cultures, Politics and Society, Turin, Piedmont 10124, Italy

**Keywords:** non-religion studies, secularity, Big Data, user profiling, text mining, automatic image classification, data harmonization, interdisciplinarity

## Abstract

The shift of attention from the decline of organized religion to the rise of post-Christian spiritualities, anti-religious positions, secularity, and religious indifference has coincided with the deconstruction of the binary distinction between “religion” and “non-religion”—initiated by spirituality studies throughout the 1980s and recently resumed by the emerging field of non-religion studies. The current state of cross-national surveys makes it difficult to address the new theoretical concerns due to (1) lack of theoretically relevant variables, (2) lack of longitudinal data to track historical changes in non-religious positions, and (3) difficulties in accessing small and/or hardly reachable sub-populations of religious nones. We explore how user profiling, text analytics, automatic image classification, and various research designs based on the integration of survey methods and big data can address these issues as well as shape non-religion studies, promote its institutionalization, stimulate interdisciplinary cooperation, and improve the understanding of non-religion by redefining current methodological practices.

## The bigger picture

It is becoming increasingly clear that secularity and non-religion are progressively turning into a relevant component of social life in both western and non-western countries and that they are connected with a variety of pressing issues such as civic engagement, human rights, and social integration. While traditional methodological approaches remain important to understand the varieties of non-religion, Big Data can significantly shape the way in which social scientists frame and analyze the puzzles within this emerging academic field. Nevertheless, large unstructured data collections and classification algorithms remain widely underused in sociology of religion, hindering its potential. We argue that to let Big Data in means to build an interconnected, interdisciplinary, and cooperative field situated at the intersection of non-religion studies and data science.

## Teaser

For decades, the scientific community collected data about religion and beliefs using questionnaires that were not developed with the issue of non-religion in mind. This is why the new field of non-religion studies faces major difficulties in opening up this category for critical empirical scrutiny. User profiling, text analytics, automatic image classification, and various research designs based on integration of survey methods and Big Data can help researchers to address these issues.

## Introduction

As Harvey Miller^1^ wrote, “the data avalanche is here. Shouldn't we be digging?” At the turn of the century, big data came with a big bang providing massive amounts of underexplored or even unexplored data, opening new horizons and making possible innovative approaches to old puzzles. As a consequence, the scientific community seized the opportunity in multiple fields ranging from artificial intelligence^2^ to medicine^3^, from biology^4^ to history,^5^^,^^6^ and so on.

Despite the rise of computational social science,^7^^,^^8^ several disciplines in sociology—such as cultural sociology or sociology of religion—showed a considerably lower propensity to take advantage of this new opportunity when compared with the so-called hard sciences or with more quantitatively oriented branches of social sciences.^9^^,^^10^ While certain areas of cultural studies recently displayed an increased interest in big data analytics,^11^, ^12^, ^13^ computational approaches to religion remain sporadic. Recent developments in the sociology of religion, despite deeply grounded fascinations with quantitative methods,^14^ have pushed multiple scholars into more qualitative directions.^15^, ^16^, ^17^ The result is that only 0.06% of 139,368 papers about religion in Web of Science databases for the years 2012–2020 explicitly engage with or make use of big data. The situation is slightly better when Scopus is considered—0.31% of 138,785 papers—but either way the percentage of studies referencing big data remain considerably limited. While quantitative methods were presented as the “methodological requirements” of the requalification of cultural phenomena both inside and outside of sociology,^10^ big data arguably represents the new frontier of neglected opportunities.

Sociology of religion is as old as sociology itself. From Weber's^18^^,^^19^ *Die Protestantische Ethik und der Geist des Kapitalismus* to Durkheim's^20^ *Les Formes Élémentaires de la Vie Religieuse*, the history of sociology goes hand in hand with the study of religion. Following the increase of spirituality studies,^21^^,^^22^ the binary distinction between religion and non-religion has lost much traction due to newly emerged research interests in opening up these categories to study (historical changes in) their composition empirically.^23^ We argue that a better integration of big data in the research on non-religion can help to accelerate the institutionalization of non-religion studies, to promote interdisciplinarity, and to address and overcome some of the most pressing puzzles in the contemporary study of non-religion.

## The spread of non-religion studies

### Beyond secularization theory

Secularization theory, once the proud theoretical flagship of sociology of religion, is one of the few theories that ever attained “a truly paradigmatic status in the social sciences”: “(It was) shared by all the founding fathers. Indeed, (…) everybody took it for granted.”^24^ This changed in the course of the 1980s, when the theory became a major target of critique,^25^, ^26^, ^27^ even though various social-scientific students of religion continued to defend it.^28^^,^^29^ Indeed, the hypothesis that more and more westerners are becoming less and less religious has meanwhile been strongly supported by empirical research.^30^ This applies even to the United States, traditionally often invoked as a counterexample to the notion of religious decline in the West.^31^

It is as such clear that the shift away from secularization theory since the 1980s has not happened in response to its empirical refutation. It is indeed precisely the other way around. For the marked decrease in numbers of those who can plausibly be characterized as identifying with Christian religion, and the increasing numbers of non-religious westerners this has resulted in, have given rise to new research questions that go beyond the religion versus non-religion binary. What does it actually mean to identify as “non religious” nowadays,^23^^,^^32^^,^^33^ not least in terms of understandings and evaluations of traditional Christian religion?^34^ And what to make of apparently increased “spirituality talk”?^35^ Questions like these have meanwhile given rise to the new fields of “spirituality studies” and “non-religion studies,” fields that both aim to go beyond the conceptual binary of religion versus non-religion to map “religion's other,” as Smith and Cragun have put it.^33^

As to increased research interests in “spirituality,” critics have identified secularization theory's notion of “religion” as in practice overly narrow, entailing basically not much more than organized religion. In doing so, they pointed out how post-Christian “self-religions” of the type that used to be called “New Age” have become more widespread in the West in precisely the same period during which Christian religion had declined,^22^^,^^36^ even though without being able to compensate for the latter.^22^^,^^29^ Self-religions of this type are rooted in western esotericism,^37^ aim at overcoming religious and secular dualism,^38^ and do as such entail a shift toward an eastern-style monistic worldview.^39^ Key research questions within the new field of spirituality studies address what people actually mean when they self-identify as “spiritual but not religious,”^35^^,^^40^^,^^41^ whether and how the composition of the category of religion has changed historically,^22^^,^^42^, ^43^, ^44^ and whether or not spirituality entails more than a “fuzzy” step in a historical trajectory from religion to “non-religion.”^45^, ^46^, ^47^, ^48^

In short, widespread ambitions of moving beyond secularization theory have led to the emergence of the field of spirituality studies, which aims to open up the religion category for critical empirical scrutiny. More recently, the new field of non-religion studies has started to do the same with the other half of the conceptual binary on which secularization theory relied—i.e., the non-religion category.^34^^,^^49^ The latter was traditionally studied in conjunction with faltering intergenerational transmission of churchgoing, informed by a conception of non-religion as a (indeed: one) residual category, diametrically opposed to religion and with its growth providing evidence in support of secularization theory.^50^, ^51^, ^52^ The new field of non-religion studies moves beyond this by instead scrutinizing (changes in) the category's heterogeneous composition, with special attention to shifts between sub-categories, such as, for example, the spiritually inclined who dismiss Christian religion; the anti-religious who dismiss religion and spirituality alike; and the religiously indifferent who despite their own non-religiousness do not object to either religion or spirituality.^34^^,^^53^ While distinctions like these are surely irrelevant in mapping the decline of Christian religion, they are absolutely vital in studying what does, and what does not, increasingly take over its former hegemony.

### The new field of non-religion studies

Early pioneers in the study of non-religion started pointing out from the late 1960s onwards that “religious nones” entailed a “neglected category” with underexplored similarities with affiliated respondents^54^ or a hardly homogeneous category in need of systematic empirical exploration.^55^ Interestingly, these early attempts at launching non-religion as a vital research area have only very recently started to pay off.

**Figure 1:**
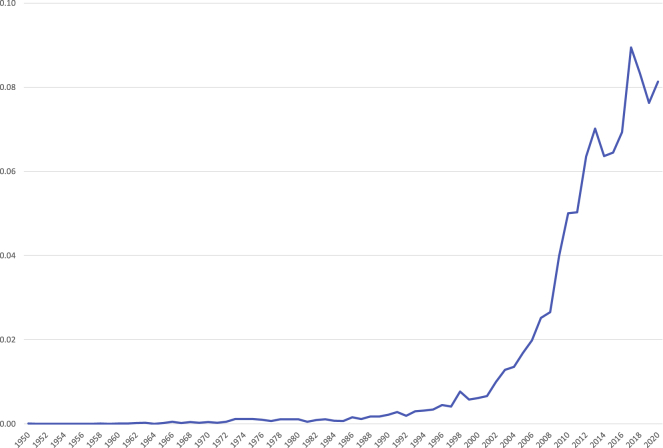
Number of scientific papers dealing with non-religion (expressed as proportion of total), Scopus 1950–2020 (N = 12,082)

The following data (see Figure 1) use the Scopus database to provide some aggregated summary statistics. Compared with Web of Science, Scopus offers a better coverage of publications within the field of social sciences and within the field of arts and humanities.^56^ The search is based on title, abstract, and author's keywords. The query first searched for the words “religion,” “religious,” or “religiosity” in contributions published between 1950 and 2020. Successively the results were refined looking for the following words or expressions: irreligion, unbelief, “non religion,” “non religious,” “no religion,” “religious nones,” agnosti∗, or athei∗.

As shown in Table 1, between 1950 and 1972 non-religion was hardly discussed by scholars within the field, with only 0.3% of the observed 12,082 papers about the topic being published during this period. Between 1973 and 1989 the debate remained substantially stagnant. The average annual production increased from 0.015% to 0.1%—i.e., an increase by 6.7 times—but remained very modest. It was during the 1990s that papers of this kind slowly started to become more common. In fact, compared with 1950–1972, the average annual production increased by more than 25 times during this period. Between 2000 and 2006 the average annual production increased even further, reaching 1.2%, and resulting in 8.6% of the total in only 7 years. This means that roughly 85% of the total scientific production about non-religion in the Scopus database was published during the past 14 years. The average annual production grew steadily during the observed period, reaching 3.8% in 2007–2011 and 7.4% in 2012–2020. A total of 66.2% of the total scientific production about non-religion, nearly two-thirds of it, was therefore published throughout the past 9 years.

**Table 1:** Average percentage per year, percentage of total and numerosity of scientific papers dealing with non-religion by period, Scopus 1950–2020 (N = 12,082)

	1950–1972	1973–1989	1990–1999	2000–2006	2007–2011	2012–2020	Total
Average percentage/year	<0.1	0.1	0.4	1.2	3.8	7.4	1.4
Percentage of total	0.3	1.8	3.8	8.6	19.2	66.2	99.9
N	41	223	465	1,038	2,321	7,994	12,082

Five decades after Vernon^54^ and Campbell,^55^ and despite the evidence of an increased attention to non-religion, testified also by the establishment of specialized research programs, such as the “Programme for the study of religion and non-religion” of the London School of Economics and Political Science or “Understanding unbelief” of the University of Kent, Nikitaki^57^ reaches a similar conclusion claiming that the focus on religious nones among scholars is still “virtually nonexistent.”

**Figure 2:**
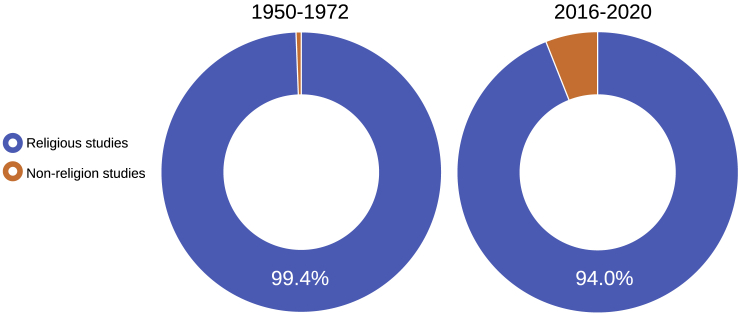
Prevalence of non-religion studies, a comparison of 1950–1972 (N = 7,093) and 2016–2020 (N = 81,176), Scopus

By looking at the sheer growth of papers about non-religion without controlling for the general trend within religious studies, there is the risk to overestimate the scope of the phenomenon. In fact, while the papers about secularity and non-religion grew over the past decades as we previously showed, the same can be said about the remaining papers with a focus on religion. As shown in Figure 2, while the prevalence of non-religion studies increased by 10 times between 1950–1972 and 2016–2020—going from 0.6% to 6.0%—the papers about non-religion still remain a minority within the broader field of religious studies. Far from being a well-established academic and scientific reality supported by a solid majority of scholars, non-religion studies thus represent an emerging field. While Web of Science shows a temporal trend that is similar to the one observed in Figure 1, compared with Scopus it tends to underestimate the share of papers dealing with non-religion.

Besides the debate about secularity and secularization discussed above, at least three other elements have arguably contributed to the growing interest in non-religion^58^: (1) the rapid growth of the population with no religious preference in highly influential countries from Europe, Australasia, Americas, and East Asia observed over the past four decades; (2) the increased acknowledgment of consistent intra-group differences of this supposedly homogeneous residual category; (3) the establishment of a normative framework sensitive to the freedom of religious or *belief* actors accompanied by the institution and recognition of associations representing religious nones—e.g., the European Humanist Federation or the American Humanist Association.

## Studying non-religion with small data

Intellectual progress in the newly emerged research field of non-religion studies is slowed down by the lack of relevant and internationally comparative survey data. This is because the large survey programs that could in principle provide such data (e.g., the European Values Study, the World Values Survey, the European Social Survey, and the International Social Survey Programme) do still rely heavily on questionnaires informed by secularization theory. The resulting data are as such perfectly useful to map the decline of Christian religion, and to some extent changes within Christianity itself,^42^^,^^44^ but they are of much less use for the study of religious change more broadly conceived,^59^ especially for historical changes in understandings of Christian religion among the waxing numbers of non-religious and for the spread of spirituality. While useful questions that are repeated across multiple waves of data collection are not completely absent, to be sure, their number is limited and by far precludes the levels of detail and precision that have traditionally been attained for Christian religion. This leaves researchers interested in the dissemination of the variegated renditions of non-religion largely empty handed, facing up to a non-religion category that has considerably increased in time, yet at the same time remains much of a black box. While the resulting “small data” do surely provide a picture of various characteristics typically associated with this black box, the collection and analysis of big data offer a major promise in overcoming the current data problems.

Nones are more frequently males than females, tend to be more concentrated in densely populated areas and to be, on average, younger than affiliated subjects.^58^^,^^60^^,^^61^ While education is traditionally positively correlated with non-religious preferences,^62^ according to Voas^52^ this trend is progressively reversing among younger cohorts in the United Kingdom. Gender, age, education, and geographical area are all typically associated with non-affiliation. Nevertheless, the predictive power of these socio-demographic characteristics was questioned,^63^^,^^64^ suggesting that the degree of worldview pluralism of local networks might play a fundamental role in the process of religious non-affiliation and disaffiliation.^63^

While the vast majority of social surveys do not allow respondents to elaborate further on their lack of religious affiliation, or distinguish only between atheism and agnosticism, recent research showed that generic non-religious labels hide a rich variety of internal differences ranging from new atheism to atheism plus, humanism, religious indifference, secularism, and so on.^65^, ^66^, ^67^, ^68^ A new comparative study of Brazil, China, Denmark, Japan, the United Kingdom, and the United States showed that only a minority of nones describe themselves as an “atheist” or “agnostic,” frequently preferring other popular labels, such as “humanist,” “free-thinker,” “skeptic,” “secular,” etc.^69^ The study also pointed out that widespread theoretical assumptions about atheists and non-believers in general, such as the strong dogmatic conviction of self-assessed atheists or the lack of supernatural beliefs among nones, are frequently violated at an empirical level.

**Figure 3:**
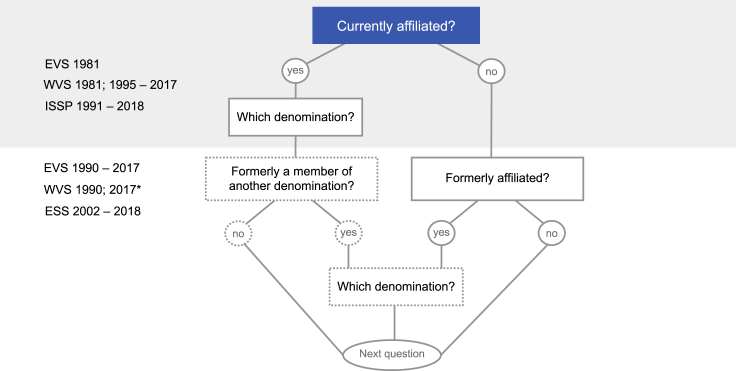
The logic of religious (non-)affiliation in the master questionnaires of the European Social Survey (ESS), the European Values Study (EVS), the International Social Survey Programme (ISSP), and the World Values Survey (WVS) ∗Between 2017 and 2020 EVS and WVS jointly collected data in several European countries following the Memorandum of Understandings.

Besides the variety of strictly non- and/or anti-religious positions, it was also evidenced that non-affiliation is not necessarily a matter of non-religiosity. According to a research conducted by Lindeman and Lipsanen,^70^ 25% of subjects with a high score on the Supernatural Belief Scale^71^ are religious nones. Frequently addressed as “spiritual seekers” or “unchurched believers,”^35^^,^^72^ these peculiar sub-categories of nones invite the research community to see religiosity beyond affiliation and to re-discuss the way secularity is both theorized and operationalized to explore the conceptual and empirical implications of the relocation of the sacred. Similar results were attained by other studies^72^^,^^73^ that found significant differences in religiosity among nones using panel data about changes in the affiliation to a religious denomination as a discriminatory variable. The authors distinguished between stable religious affiliates (respondents who were affiliated at both of the considered time points), liminal nones (respondents whose affiliation changed during the observed period), and secular nones (respondents who were unaffiliated at both of the considered time points). Liminal nones tend to waver between religion and non-religion and were found to be, on average, more religious than their secular counterparts.^72^^,^^73^ However, the application of this distinction in cross-sectional studies that researchers commonly use is problematic because the resulting classification strategy is not sufficiently fluid. As shown in Figure 3, the questionnaires of major international social surveys do not tell much about the meaning of non-affiliation and offer limited information about conversion and deconversion trajectories. A considerable number of these surveys (top half of the figure) register only the current state—usually relying on general labels, such as “none,” “no religion,” or “none of the above”—without follow-up questions other than affiliation at the age of 12. The European Social Survey and more recent waves of the European Values Study (bottom half of the figure) represent an exception in this sense. Despite minor variations, both surveys ask nones whether they formerly belonged to a religious denomination. In 2008, affiliated European Values Study respondents were also asked whether they previously belonged to a different denomination. Starting from 2017, the European Values Study no longer asks which religious denomination disaffiliated nones formerly belonged to. In Figure 3, the minor coverage of these questions is represented by dashed contours. Affiliates are typically not asked whether they used to be non-religious at some point of their life, a characteristic that would make them liminal nones rather than stable affiliates. Moreover, stable non-affiliation does not necessarily exclude spirituality, whether inspired by religious traditions^42^ or secular alternatives.^74^^,^^75^

Currently, the field of non-religion studies is facing at least three major obstacles. First, there is the well-known overabundance of labels employed to address nones and non-religion in the scientific literature.^76^^,^^77^ It is important to contain the creative impulse of social researchers in favor of a systematic reorganization of the existing corpus of knowledge. Such an effort is crucial for a sustainable interdisciplinary cooperation and constitutes the prerequisite of clear, explicit, and testable theoretical statements. Nevertheless, the terminological issue is not just a matter of theory. Wording matters, which leads to the second issue faced by social researchers. As testified by the case of the UK census of 2011—which replaced the term “none” with “no religion”—different labels can significantly affect survey results.^78^^,^^79^ To develop alternative classification strategies of religious non-affiliation it is thus important to go beyond binary choices resting on a misleading understanding of the no religious preference^35^^,^^80^ and to study the effect of different labels on respondents' (non-)religious self-identification. The third obstacle concerns the availability of suitable data to test emerging hypotheses about non-religiosity, individualized religion or New Age spiritualities. Not only the questionnaires of international surveys are considerably biased toward traditional western religiosity,^81^ frequently neglecting the variety of eastern beliefs and their implications for individualized religion, post-Christian spirituality and religious bricolage,^82^ but they rarely cover supernatural, non-religious, or anti-religious preferences.

Survey methods can be improved and new variables included to cover neglected aspects of the contemporary religious and non-religious landscape. It will be costly and time consuming, but several scholars are heading in this direction, as testified by the widespread critique of the predominant survey model,^59^^,^^67^^,^^78^^,^^79^^,^^80^^,^^83^^,^^84^ by the arrival of dedicated surveys,^69^^,^^85^^,^^86^ by the development of new measurement methods,^47^^,^^87^ or by the progressive institutionalization of non-religion studies.^88^ This will significantly improve researchers' capacity to analyze the multifaceted reality of non-affiliation today, but it will leave their ability to explore past historical trends—which is a crucial aspect in the debate about secularization—largely unaffected.

Another potential limit is represented by the numerosity of these groups. Religious nones grow in numbers and constitute a stable majority in several countries, such as the Czech Republic, France, Great Britain, Hungary, Japan, or the Netherlands, but once the scientific community opens the Pandora's box of religious non-affiliation to have a closer look, this solid majority will be fragmented into a multitude of smaller realities. Some of them will be big enough to avoid large confidence intervals, but other groups will be considerably smaller or just difficult to reach with small N studies.

In 1981, when the European Values Study and the World Values Survey started their very first fieldwork, the percentage of nones in participating countries was on average 11.3%. Today the most recent data of the same two surveys reveal that the overall percentage of nones has doubled, and that it tripled in Europe. As of 2020, nones represent the new majority in the Czech Republic, Estonia, France, Hong Kong, Hungary, Japan, Macau, the Netherlands, Great Britain, South Korea, and Vietnam, ranging between 54.1% and 86.8%. Australia, Belgium, Colombia, New Zealand, and the United States are already approaching the 50% threshold, while several other countries are heading in the same direction.^58^ In the meanwhile, researchers became increasingly aware of relevant within-group differences in terms of, for example, beliefs,^69^^,^^72^^,^^73^ civic engagement,^89^ or political orientation,^90^ recognizing secularity as a socially relevant issue. We argue that, by letting big data in, non-religion studies scholars can exploit a variety of novel sources of data to improve coverage (in both time and space), granularity, and predictive power while containing costs. Furthermore, a major opening to quantitative methods and big data analytics will positively affect also the interdisciplinarity of this emerging field and speed up its institutionalization.

## Studying non-religion with big data

In recent years, the discussion about big data kept rapidly growing, not without a considerable level of hype among both scholars and business professionals. According to a popular—and strongly criticized^91^, ^92^, ^93^—narrative, big data came to revolutionize everything making previous science obsolete.^94^^,^^95^ More recently, rather than suggesting replacing small data with big ones, researchers argued that integration between these two methodologies or “cultures of modelling”^96^ is both possible and necessary.

In the following, the promises, limitations, and potential pitfalls for non-religion studies of user profiling, text analytics, automatic image classification, and various research methods mixing small and big data will be considered to discuss some of the most promising routes made available to scholars by recent developments in data analytics.

### User profiling

User profiling leverages predictive analytics and machine learning to infer a variety of individual-level information, ranging from personality traits to political preferences, religion, and behavioral habits. Whether based on explicit and/or implicit techniques, user profiling is a grounding element of modern service personalization.^97^ Following the large-scale diffusion of smartphone devices, a rich variety of user-generated data were employed for this purpose: call logs, messaging logs, lists of installed apps, app usage patterns, and other mobile phone data.^98^, ^99^, ^100^, ^101^ The usage of social media (e.g., Facebook, Twitter, Instagram, etc.), the browsing activity and website navigation, credit card transactions, etc., constitute other examples of data frequently used in user profiling.^102^, ^103^, ^104^

For example, Nguyen and Lim^105^ have employed Support-Vector Machine classifiers to predict religion labels of users based on microblogging data collected from Twitter users in Singapore. Given the small numbers of self-declared Buddhist, Taoist, and Hinduist users, the authors focused on Christian and Muslim respondents, showing that the proposed classification strategy can accurately predict religious affiliation by combining textual features and social links.

Gathering labeled data to create training samples is not an easy task, but gathering labeled data about users' religious non-affiliation is arguably even trickier. An approach called “co-labeling” was developed to deal with ambiguous problems where labels of users in the training sample are uncertain.^106^^,^^107^ Co-labeling is basically a multi-view learning method that combines classifiers trained on different views to improve the effectiveness of unlabeled data by allowing different classifiers to inform each other.

Privacy concerns^108^ and the effective availability of usable data^91^ to the researchers are potential obstacles to a research design relying on co-labeling to develop an efficient classification strategy of religious nones. Regarding this, further limitations, at least from a sociological standpoint, are certainly represented by potential sampling/selection bias due to the access to limited segments of the population (e.g., the ones present and active on social media platforms, the owners of smartphones) as well as the relatively limited time span accessible through this particular approach. User profiling relies on large-scale diffusion of big data and does not provide means to reconstruct detailed historical trends for the past century.^109^ Nevertheless, co-labeling constitutes a powerful technique that uses massive amounts of data generated by users on a daily basis that could be used to train and employ dynamic classification strategies of users' latent attributes, such as religious non-affiliation. This can greatly improve the knowledge of the varieties of non-religion—effectively assisting researchers in their effort to deconstruct the black box of non-religion—as well as the access to outliers and minorities,^110^ minimizing uncertainty and avoiding large confidence intervals. While the latter categories can be studied through a variety of ethnographic and qualitative techniques integrating the current theoretical framework, an approach based on big data can significantly accelerate the process at the international level. Furthermore, the results can be used in correlational studies of institutional trust or political participation, and to refine classification strategies employed by international social surveys.

### Text analytics

At least since the late 1970s, researchers' toolkits contain rudimental text-mining techniques to parse unstructured textual data and identify patterns.^111^ Nevertheless, with the improvement of natural language processing and machine learning techniques, and with the large-scale diffusion of unstructured data, text mining underwent a rapid development in computational sociology and digital humanities. Specifically, topic modeling and sentiment analysis are of particular interest for non-religion studies scholars.

Topic modeling typically uses Latent Dirichlet Allocation (LDA), Latent Semantic Analysis (LSA), or Probabilistic Latent Semantic Analysis (PLSA) to analyze large and unstructured collections of textual documents in a longitudinal and/or comparative perspective to identify recurrent topics and latent semantic structures.^112^^,^^113^ While a classical clustering algorithm returns one label to characterize a document (e.g., k-means clustering), with topic modeling it is possible to analyze a probabilistic composition of the text. Sentiment analysis, on the other side, is constituted by a series of techniques—based on natural language processing—used to study affective states, opinions, and other subjective information conveyed by a text.^114^^,^^115^ While automated approaches to text still struggle to adequately frame sarcasm and irony,^116^ posing relevant methodological concerns, topic modeling and sentiment analysis can be combined^117^ to compare and contrast the characteristics of non-religious discourses in time and space.

While big data may be young, meaning that it is hard to provide the analysis with a long-term longitudinal dimension, “big data of the past”^118^ are potentially as old as humanity itself and offer a precious source of under-analyzed information. For example, magazines published periodically and explicitly intended for an atheist, humanist, and/or secular audience cover over a century of modern and contemporary history. Among others: *New Humanist Magazine* (United Kingdom, 1885–current), *The Humanist Magazine* (United States, 1941–current), *The Skeptic* (Australia, 1981–current), *Les Cahiers Rationalistes* (France, 1930–current), *Espace de libertés* (Belgium, 1980–current), *L'ateo* (Italy, 1996–current—renamed as *Nessun Dogma:Agire laico per un mondo più umano* in 2020).

The main advantage of this approach is the availability of rich and long-term historical data that non-religion studies are currently missing: it combines nominally available data with recent advancements in text analytics to extrapolate new value from old information by analyzing the changes over time in non-religious discourses, but also national specificities and international differences. In this sense, an integration of big data and modern computational techniques in current research practices might be the only way for social scientists to historically trace the development of varieties of non-religiosity.

However, the application of text analytics to secular magazines may imply careful and time-consuming archival work to digitize older issues and prepare a dataset for the analysis. The necessary pre-processing can be reduced by reorienting the focus on readily available material (e.g., tweets, Reddit comments, dedicated forums), but this will also considerably decrease the time window of the analysis undermining one of the main advantages of this particular approach for non-religion studies. Books on atheism, secularity, or non-religion represent another alternative source of information. Nevertheless, compared with atheist magazines, books constitute a very heterogeneous group of materials—ranging from philosophical treatises to essays, science fiction, commentaries, and more—which are not always easily comparable. For this reason, and because of the temporal regularity of publications, magazines arguably represent a better proxy of non-religious discourses in time.

A second limitation is imposed by the very nature of books and magazines, which implies a form of institutionalization of the non-religious position through the editorial board that might or might not reflect accurately the social reality, thus making marginal, indifferent, and/or “liminal” positions^73^ particularly difficult to observe with this approach. Once again, shifting attention to other sources, such as Twitter or Reddit, represents a valid alternative that solves this issue but poses its own problems—namely, the risk of exchanging highly active and vocal minorities for something much bigger.

Multipurpose international social surveys started to appear in the early 1980s, but were initially confined prevalently to Northern America and Western Europe. It was only a decade later that these survey programs started to improve their reach in other geographical areas, but even leaving aside persisting gaps in Asian and African data, two problems remain: (1) harmonization of different waves of the same survey or of different surveys is a problematic process.^119^ In this sense, increasing the time horizon or extending the observation to a larger group of countries typically comes at the cost of a considerable decrease of comparable variables; (2) and, more importantly, questionnaires of large survey programs remain theoretically informed by secularization thesis and keep struggling with contemporary religious and non-religious landscapes.^59^ While the field of data harmonization is a rapidly expanding sector significantly improving the quality of newly collected data, new surveys, such as Understanding Unbelief,^69^ the Secular Voices Survey,^85^ or the Secular Communities Survey^86^ tackle the latter issue. Nevertheless, future waves of dedicated surveys remain uncertain, coverage of countries is still considerably limited and data are currently not public. Most importantly, in both cases the quality of existing longitudinal data is suboptimal for the study of non-religion. Once magazines and other historical sources are fully recognized as data, it becomes possible to considerably extend the accessible time horizon without necessarily sacrificing the depth of the analysis. Potential applications of text analytics ranges from longitudinal designs of the kind described above to comparative studies of local groups or meta analyses of the scientific community studying non-religion.^120^ Due to the massive increase of availability of unstructured textual data observed throughout the past two decades, text mining represents an important tool in the future study of non-religion.

### Automatic image classification

While text clearly constitutes a relevant part of the unstructured data available today, images are another precious source of information. The use of visual methods in social sciences is hardly new. In 1998, Homer presented the “visual culture” as a new paradigm^121^ in research. Over the years multiple studies applied visual methods, for example: Peraica studied contemporary forms of self-representation analyzing selfies,^122^ Arbulla and Bucchi focused on warning messages posted in public spaces,^123^ Vindrola-Padros used drawings to study the daily life of children in Argentina,^124^ and Eide analyzed preschool Christmas holiday specials to understand the depiction of religious holiday practices in the United States.^125^ What all these very different studies have in common is a qualitative approach to the analysis of visual data. While qualitative approaches to visual culture are still more appealing to social scientists, the increased availability of large collections of visual data and the development of innovative artificial intelligence approaches and tools force the scientific community to consider possible strategies to implement automated methods in the research process.

So how can computational science help researchers interested in the analysis of images? Arguably, at least two general strategies can be defined. The first strategy is to use automatic image classification to reduce the complexity of large data collections by extracting a small but highly consistent cluster of visual objects with similar characteristics to analyze with qualitative techniques. The researcher might want to distinguish, for example, between photographs and graphics,^126^ between scenes occurring indoor and outdoor,^127^ between portraits of men and women,^128^ between images with and without people, or between photos of individuals and groups.^129^ In addition, following the contribution of computer vision to the analysis of artworks, a weakly supervised object detection approach could be implemented to identify, distinguish, and classify various iconographic elements^130^^,^^131^ observable in the considered images. In principle, a researcher can apply multiple criteria to progressively reduce the number of cases into a sub-set of theoretically relevant, conceptually consistent, and qualitatively manageable objects. Alternatively, if the resulting selection is still too big, sampling methods can be used to further reduce the number of observations. The second general strategy uses similar clustering techniques and the available metadata but, instead of extracting a specific cluster of images to analyze qualitatively, it focuses on the totality of the resulting clusters—or on a selection of relevant clusters—from a quantitative standpoint. Projects of this kind can analyze visual signatures of geographical areas exploring spatial and temporal visualizations of images,^132^ study the physicality and visual content of cultural production,^133^ or explore identity and self-representations of users on social media.^134^^,^^135^

The choice of the strategy will depend on available resources and on the research question, but the major limitation of this approach is arguably represented by the limited availability of large thematic collections about non-religion. So what kind of visual data might non-religion studies' scholars be interested in? In the previous section we considered the use of textual data from a variety of sources, such as secular popular press, books, or dedicated forums. However, these sources are usually not limited to textual data. The covers of books and magazines, as well as the visual content that they convey, can become a relevant part of the analysis of visual culture in organized non-religious groups. Communication strategies of non-religious communities could be analyzed using the visual content related to campaigns of various secular associations around the globe—such as the recent campaign of the *UK Humanist* asking non-religious people to answer “no religion” in the upcoming British census or the campaign of the Italian Union of Rationalist Agnostics and Atheists for the legalization of debaptism. Discussions generated on dedicated forums, such as “atheist forums,” “think humanism,” or “the agnostic forum” are frequently accompanied by the production and/or sharing of graphical objects, for example, memes, that can be used to study secular representations of religious others. Conversely, religious representations of atheism or secularity could be approached in a similar way. A final example of potentially interesting sources of visual data about non-religion is represented by social media. In particular, Twitter and Instagram could be used to analyze co-occurrences of popular non-religious hashtags and characteristics of visual objects associated with them. In principle, this could be extended to religious communities in a comparative analysis of religious, non-religious, and anti-religious self-representations.

### Mixing small and big data

Mixing small and big data, rather than a specific line of investigation, constitutes an umbrella of possible approaches based on the manipulation of the observed situation and/or on the possibility of integrating big data with survey methods or various qualitative techniques directly.^136^

The relevance of mobile applications for user profiling was already discussed above. While mobile data pose considerable difficulties in terms of access, scientists around the world have started to design applications to combine mobile data with survey methods to address research topics ranging from time allocation^137^ to academic performance,^138^^,^^139^ behavioral habits,^98^^,^^101^^,^^140^ or mental well-being.^139^^,^^141^ The possibility of designing mobile apps to collect relevant information about non-religion and to cross-reference it with survey data to analyze non-religious preferences, behavioral patterns, and more, offers multiple advantages and possibilities.

The current attempt to redefine survey methods in non-religion studies is accompanied by severe coverage issues, with relevant data being collected for only a minority of countries at a single time point and being still mostly unavailable to the wider scientific community. An increased usage of big data offers the possibility to fill the existing gap of relevant information considerably faster and at a lower cost. While the belief that big data is replacing rather than integrating survey methods still permeates computational sciences,^95^^,^^142^ it is increasingly more and more common to encounter examples of research relying on a combination of big and small data.^98^^,^^143^ The resulting reflections on commonalities and potential integrations^96^^,^^144^ of heterogeneous data sources—whether structured, semi-structured, or unstructured—arguably represent one of the major challenges in the contemporary field of data harmonization.^145^^,^^146^

Merging public, private, and governmental sources of data to produce a multidimensional dataset is also another way to approach otherwise hardly accessible clusters of population, such as, for example, people with a vegetarian lifestyle.^143^ The decision tree learning algorithm employed by Lusk^143^ offers additional insights and can outperform logit and regression models by fully exploiting the size and complexity of big data. This research design thus overcomes the problem of small or hardly accessible groups^110^ frequently experienced in non-religion studies and might be useful to develop a classification tree of religious nones and to monitor the impact of (non-)religious preferences on their decision making.

## Final considerations

Religious nones and non-religion play a fundamental role in sociology of religion and in the emerging field of non-religion studies. A big data-based approach to the topic can greatly improve our understanding of the phenomenon in contemporary societies, of its evolution over time, and of relevant cross-national specificities and differences. This is certainly a major point of concern for sociology of religion and non-religion studies—which can in turn accelerate its institutionalization within sociology of religion and further support its emerging theoretical interests through an increased big data usage. Nevertheless, the implications of an improvement in the scientific study of non-religion go beyond sector-specific theoretical interests: social and institutional trust, political participation, political orientation, civic engagement, business strategies, palliative care, general patient care, and social integration^89^^,^^90^^,^^147^, ^148^, ^149^, ^150^, ^151^, ^152^, ^153^, ^154^ are, in fact, some of the research areas frequently associated with religious (non)affiliation. These fields can thus greatly benefit from an improvement in the parallel field of non-religion studies by further exploring empirical implications of a better understanding of religious nones.

The proposed approaches tackle some of the more pressing issues currently faced by scholars in this emerging field: development and implementation of alternative classification strategies of non-religion; lack of coverage of relevant variables in international surveys; lack of detailed longitudinal data; and access to small and/or hardly reachable sub-populations of religious nones.

The prefigured outcome, as well as the proposed methodological strategies, presupposes analytical approaches that go well beyond the currently dominating techniques in sociology of religion. As such, the success of the operation might require the establishment of an interconnected, cooperative, and interdisciplinary sociology of non-religion integrated not just within the broader context of sociology of religion, but also in the rapidly expanding fields of computational social sciences, digital humanities, data visualization, and data harmonization.

### Resource availability

#### Lead contact

Further information and requests for resources and materials should be directed to and will be fulfilled by the lead contact, Dominik Balazka (dominik.balazka@unimi.it).

#### Materials availability

This study did not generate any new material.

#### Data and code availability

The summary statistics illustrated in this paper are based on data available in Scopus (https://www.scopus.com/), for a description of the procedure see section 2.2. The authors declare no unpublished custom code, software, or algorithm.
